# What explains the provision of health insurance by Indonesian employers? A trend analysis of the National Labour Force Survey 2018-2022

**DOI:** 10.1093/heapol/czae053

**Published:** 2024-06-20

**Authors:** Levina Chandra Khoe, Muchtaruddin Mansyur, Virginia Wiseman, Augustine Asante

**Affiliations:** School of Population Health, University of New South Wales, Sydney, NSW 2052, Australia; Department of Community Medicine, University of Indonesia, Jakarta 10310, Indonesia; Department of Community Medicine, University of Indonesia, Jakarta 10310, Indonesia; Kirby Institute, University of New South Wales, Sydney, NSW 2052, Australia; School of Population Health, University of New South Wales, Sydney, NSW 2052, Australia

**Keywords:** Employers, health insurance, occupational health

## Abstract

Indonesian laws mandate that every employer should provide health insurance and work accident insurance to their employees. Nevertheless, there is a significant gap in the coverage of employer-sponsored insurance among Indonesian workers. This study examines the coverage of employer-sponsored insurance and work accident insurance and analyses the characteristics of the uninsured working population in Indonesia. We analysed nationally representative cross-sectional data from the National Labour Force Survey (NLFS) conducted between 2018 and 2022. The primary dependent variable was the provision of health insurance and work accident insurance by employers. The independent variables included having any physical disabilities, number of working hours, duration of employment, labour union membership, earning at least the provincial minimum wage, having a written contract and working in high risk jobs. Logistic regression was employed using the R statistical software. The findings indicate that coverage of employer-sponsored health insurance is low in Indonesia—ranging from 36.1% in 2018 to 38.4% in 2022. Workers with a written contract, earning at least the provincial minimum wage, were members of a labour union, employed for at least 5 years and working more than 40 hours a week were more likely to be insured. By contrast, workers who had physical disabilities or were employed in high-risk jobs were less likely to be insured. Our study concludes that having a written employment contract is the single most influential factor that explains the provision of employer-sponsored health insurance in Indonesia. The country’s labour laws should therefore formalize the provision of written employment contracts for all workers regardless of the type and nature of work. The existing laws on health insurance and work accident insurance should be enforced to ensure that employers meet their constitutionally mandated obligation of providing these types of insurance to their workers, particularly those engaged in high risk jobs.

Key messages

- Over the 5-year period from 2018 to 2022, coverage of employer-sponsored health insurance remained low in Indonesia, averaging around 37.1%.
- Employees with written employment contracts, earning at least the provincial minimum wage, were members of a labour union, employed for at least 5 years and were working more than 40 hours a week were more likely to be insured by their employers compared with those without contracts, who were earning less than the minimum wage, not affiliated with a labour union, employed for less than 5 years and working less than 40 hours per week.
- The majority of uninsured workers were engaged in casual work arrangements and worked in high-risk jobs.
- There is an urgent need to expand the coverage of employer-sponsored health insurance by formalizing the provision of written employment contracts for all workers and enforcing the existing labour laws to increase employers’ compliance.

## Introduction

Everyone has the right to social security; this means every individual has the right to be protected against poverty and social exclusion in the event of unemployment, maternity, injuries, sickness, disability and old age throughout the life cycle (Razavi, 2022). The right to social security is enshrined in numerous human rights laws and constitutions, such as the Universal Declaration of Human Rights and the International Covenant for Economic, Social, and Cultural Rights (UN, 1948; OHCHR, 1966). Social security, through cash or in-kind payments, is designed to ensure individuals and families have access to essential healthcare and basic income support.

The International Labour Organization (ILO) is committed to achieving social security for all workers by developing strategies as outlined in the ILO Social Protection Floors Recommendation 2012 (No. 202) (ILO, 2012). One of the strategies is to provide various social benefits at the national level, such as unemployment benefits, sickness and healthcare benefits, employment injury benefits, disability benefits and old age benefits (ILO, 2012). The benefits can be administered through either a non-contributory or contributory scheme. In a non-contributory scheme, beneficiaries do not contribute to paying the premium, while in a contributory scheme, members contribute a share of their income towards the payment of the premium.

Globally, about 46.9% of the working population is protected by at least one social security benefit scheme, and only 30.6% is covered by a comprehensive system that covers full range of benefits including child benefits and pensions (ILO, 2021b). The biggest coverage gaps are in Africa, Asia and the Arab states (ILO, 2021c). Specifically, in the Asia-Pacific region, only 38.9% of the population is effectively covered by at least one social security benefit scheme (ILO, 2021c). In many low- and middle-income countries (LMICs), providing social security is challenging as the majority of the working population is in the informal sector or works in non-standard employments such as temporary, casual or part-time workers (Winkler *et al*., 2017). More than half of all workers in Southeast Asia and the Pacific region are engaged in informal sector employment, which usually comes with limited access to social security benefits (Winkler *et al*., 2017; ILO, 2021b).

Workers face a wide range of occupational hazards in their workplace, including biological, physical, chemical, ergonomic and psychosocial hazards, that could lead to various work-related diseases or injuries (WHO/ILO, 2021). Globally, almost 2 million deaths occur annually due to work-related diseases and injuries (WHO/ILO, 2021), which could lead to an estimated economic loss of 1.8–6% of gross domestic product (GDP) (Takala *et al*., 2014). These costs are expected to be covered through work accident insurance; however, about 70% of workers worldwide do not have any insurance to cover these costs (ILO, 2021b). A lack of social security systems could lead to an unhealthy workforce who are susceptible to social and economic insecurity.

The Indonesian Law on Social Security for Workers mandates that every worker has the right to social security, which encompasses work accident insurance, death benefits, old age benefits, pension, unemployment benefits and health insurance (GoI, 1992). The workers herein include those who receive salary from employers (i.e. wage earners or *Pekerja Penerima Upah—*PPU) and those who carry out their business independently (non-wage earners or *Pekerja Bukan Penerima Upah—*PBPU). For workers employed by non-state employers, the social benefits are provided by the Social Security Agency for Workers (*Badan Penyelenggara Jaminan Sosial Ketenagakerjaan—*BP-Jamsostek). There are other agencies, such as the Civil Servant Insurance Savings (TASPEN) who managed social insurance for civil servants, and the Indonesian Armed Forces Social Insurance (ASABRI) who arranged it for military personnel. The social insurances referred here include the work accident insurance, death benefits, pension, old age benefits and unemployment benefits. However, the health insurance is coordinated by the Social Security Agency for Health (*Badan Penyelenggara Jaminan Sosial Kesehatan—*BPJS-K) under the National Health Insurance (*Jaminan Kesehatan Nasional—*JKN) scheme.

Work accident insurance (*Jaminan Kecelakaan Kerja*—JKK) is provided to cover work-related injuries and diseases. For wage earners, the JKK is covered solely by employers, in which they have an obligation to pay the monthly premiums to BP-Jamsostek, ranging from 0.10% to 1.60% of the employee’s salary, depending on the hazard risk in the workplace (GoI, 2023). The higher the risk for occupational hazards, the higher the rate of premium that would be applied. Premiums for the non-wage earners (PBPU) are adjusted to average monthly income as stipulated in President Regulation number 64 year 2020. The JKK insurance would cover the medical expenses, income replacement benefits and compensation for permanent impairment, if any.

For diseases not related to work, the workers are covered through the JKN scheme. JKN premiums are either paid by employers on behalf of their workers or paid independently in the case of non-wage earners (PBPU). The premium is set at 5% of employee’s monthly salary, for which the employer contributes 4% and the employee 1%. The JKN provides cover for the cost of outpatient and inpatient medical treatments, as listed in the benefit package.

In 2022, BP-Jamsostek reported that only about 40% of the Indonesian working population was covered by work accident insurance (BPJS-TK, 2022). The effective coverage rate, measured by the number of actively registered workers divided by the total workforce, was even lower, at around 26.5% (BPJS-TK, 2022). Thus, more than two-thirds of the working population were not covered by any insurance. Even though membership of work accident insurance is compulsory for employers and their employees, the coverage is still low. The determinants of enrolment in social security schemes are multi-faceted. Individual factors, such as age, educational level, residency, marital status, earnings, are reported to be significantly associated with health insurance enrolment. Aside from individual determinants, there are also factors from social security systems, e.g. inadequate efforts to raise public awareness, lack of trust in the agencies’ capability to manage the fund, lack of priority investment into workers’ safety and health and weak enforcement of the relevant labour laws (ILO, 2018; 2021a). Moreover, the vast majority of workers in Indonesia is engaged in small and medium-sized enterprises, where they are more likely to experience high employment turn over, to have job insecurity and to be underpaid.

Despite the low coverage, there is limited knowledge of the characteristics of the uninsured and the factors influencing their insurance status. This paper aims to examine the coverage of employer-sponsored health insurance and work accident insurance among Indonesian workers and analyse the characteristics of the uninsured working population using data from five rounds of the National Labour Force Survey (NLFS) 2018–2022.

## Methods

We performed a secondary data analysis using data from five rounds of the National Labour Force Survey: 2018–2022. The NLFS is a nationally representative cross-sectional household survey conducted biannually by Indonesia’s Central Statistics Agency (Badan Pusat Statistik, BPS). Every round is divided into two waves of data collection, with the first wave administered between January and February and the second conducted between July and August. In both waves, the survey is administered face-to-face by enumerators from the BPS.

Each round of the BPS has adopted a three-stage stratified sampling approach. The sampling procedure involved three stages: in the first stage, a list of census blocks was developed from the 2010 population census, with information on the number of households and their urban/rural classifications. A census block in Indonesia is defined as an enumeration area in a sub-district area and comprises around 100 households (BPS, 2015). The second stage involved selecting 40% of the population census blocks using probability proportional to size, with the size of the number of households and type of business field. The third and final stage involved the selection of households from each census block using a systematic sampling approach (BPS, 2019a).

The first wave of the survey is designed to collect data at the provincial level, whereas the second wave focuses on the district level. Thus, the number of census blocks selected in the first wave differed from those in the second wave of the survey. In the first wave, 7500 census blocks were selected from 30 000 district/city census blocks. The total sample for the first wave was 50 000 households for 2018 and 75 000 households for 2019 to 2022. In the second wave, 30 000 census blocks, consisting of 7500 for the first wave and an additional 22 500, were selected to obtain the estimated number of households for the district level. The total sample size for the second wave survey was 300 000 households taken in August each year. The overall participation rate of the NLFS is over 90% (BPS, 2018; 2019b; 2020).

This analysis uses data from the first wave of the NLFS from 2018 to 2022, except for 2021, where we used data from the second wave. Data for the outcome variable (employer-sponsored health insurance) were not available in the first wave of the 2021 NLFS. The sample size of the second wave of the NLFS is much larger than the first wave. To make the 2021 NLFS second wave data comparable with the sample size from 2018–2020 NLFS first wave data, we adjusted the data from the second wave. We did this by randomly selecting a comparable sample from the second wave of the 2021 survey, based on the estimated rate of increase in the insured and uninsured population from the 2018–2020 surveys. A detailed explanation of this adjustment can be found in Appendix 1.

### Study population

The NLFS covers the working-age population in Indonesia. We defined the working-age population as those in the productive age range of 15–64 years. In addition, the term working population describes those who are currently engaged in economic activities, either paid or unpaid, for at least 1 hour in the past week (BPS, 2019a). Descriptive statistics were used to generate an overview of the employment characteristics of the working-age population. Bivariate and multivariate analyses were employed to analyse factors contributing to the provision of employer-sponsored health insurance to the workforce, which include the following: permanent/contract employees, casual employees in the agricultural sector, and casual employees in the non-agricultural sector. The regulation to provide employer-sponsored health insurance to workers applies only to these groups.

### Outcome variable

The main outcome variable in this study was employer-sponsored health insurance (ESHI). We defined this as the percentage of permanent/contract employees and casual employees sponsored for health insurance and work accident insurance (JKK) by their employers. The term health insurance in this context includes the provision of JKN, private health insurance, reimbursement of medical costs by employers or the provision of health services at the workplace. This definition of health insurance is aligned with the survey guide of the National Labour Force Survey (NLFS). Further analysis to obtain an estimate of JKK coverage was also conducted.

### Exposure variables

The exposure variables comprised a range of individual and occupational-related variables, including education level, age, gender, marital status, employment status, physical disabilities, number of working hours, duration of employment, union membership, monthly wage, written contract and job risk category. Education was sub-categorized into two groups: above and below high school. Employment status was divided into several categories: (1) self-employed with no assistant; (2) self-employed assisted by family members/unpaid workers; (3) self-employed assisted by paid workers; (4) permanent/contract employees; (5) casual employees in the agricultural sector; (6) casual employees in the non-agricultural sector; and (7) unpaid/family workers. Physical disability was defined as having at least one of the following impairments: visual/hearing/speech/mobility impairment. Union membership status refers to being registered as a member of the labour union (regardless of whether membership was active or expired). The number of weekly working hours was divided into two categories: below 40 hours and 40 hours or above. The cut-off of 40 hours per week was used because this is the regular weekly working hours based on Indonesian Law Number 11 Year 2020 (Indonesia, 2020). Duration of employment was sub-categorized into working for 5 years or more and working for less than 5 years. Monthly wage was categorized into earning at least the minimum provincial wage or above and below the minimum provincial wage (BPS, 2020). Job risk category was defined based on the potential risk of work-related injuries or diseases. BP-Jamsostek categorizes occupational risk into five categories: level I (very low risk); level II (low risk); level III (moderate risk); level IV (high risk); and level V (very high risk), based on the type of occupation and business field (Indonesia Ministry of Labour, 2015). We regrouped these into three categories by combining level I and II into low risk, level IV and V into high risk, leaving level III unchanged as moderate risk. Mining, construction, transportation and shipping were categorized as high risk occupations. Agriculture, fishery, forestry, trading, automotive reparation, electricity/water/gas and waste management were included in the moderate risk group. Finance, communication and information, education, health care and other service industries were deemed low risk occupations. In the logistic regression, we aim to understand whether high risk jobs influence the coverage of employer-sponsored health insurance; thus, we further regrouped the risky jobs into two categories: high risk and other risks, defined as low-to-moderate risk jobs. In the logistic regression, we analysed the following independent variables: duration of employment (0 = less than 5 years; 1 = at least 5 years); working hours per week (0 = 40 hours or less; 1 = more than 40 hours); labour union of membership (0 = not being a member; 1 = being a member); high risk job (0 = low or moderate risk; 1 = high risk); employment contract (0 = not having a contract; 1 = having a contract); physical disabilities (0 = not having any physical disabilities; 1 = having at least one physical disabilities); and monthly wage (0 = earning less than provincial minimum wage; 1 = earning at least provincial minimum wage).

### Data analysis

Descriptive statistics were used to summarize all variables and present them in a table. Logistic regression analysis was performed to estimate the odds ratios with 95% confidence intervals (CI) for being insured vs not being insured. In the first logistic regression analysis, the outcome variable was employer-sponsored health insurance (ESHI), covering either health insurance or work accident insurance. In the second analysis, we focused on only employer-sponsored work accident insurance (JKK). We carried out this analysis to specifically observe employers’ compliance with the government regulation on work accident insurance as this is tied more to the workplace than general health insurance. In both analyses, missing data were treated as missing if they exceeded 1% but were included in their natural continuous form if they were less than 1%, as they were assumed to be insignificant. Sampling weights from the surveys were used to ensure our study population reflects the overall population of Indonesia. We assessed the interaction term between the dependent variables and plotted the model to predict insurance coverage with and without the interaction term. All analyses were conducted using the R statistical software.

## Results

**Figure 1.**
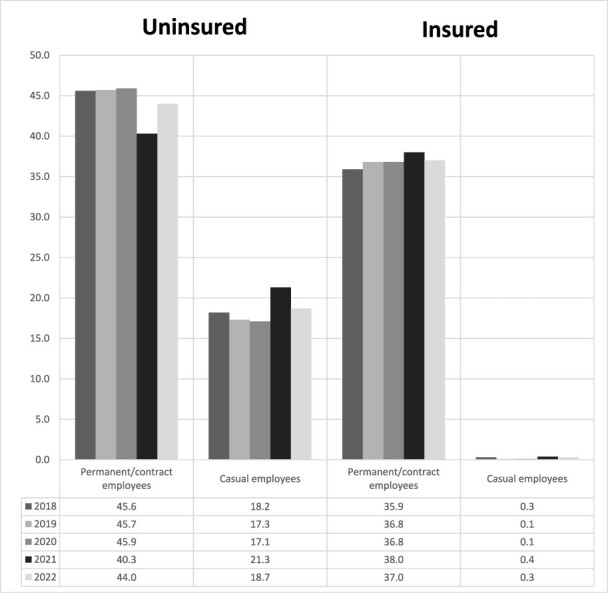
The trend of insurance coverage among the Indonesian workforce from 2018 to 2022 based on employment status

The results indicate that the average coverage of employer-sponsored health insurance (ESHI) among Indonesian workers is 37.1%. The ESHI coverage from 2018 to 2022 was 36.1%, 36.9%, 36.9%, 38.4% and 37.3%, respectively. Figure 1 shows the trend of insurance coverage among the Indonesian working population from 2018 to 2022 based on employment status (permanent/contract employees vs casual employees).

Male employees, employees who were married and those with education levels above high school dominated the Indonesian workforce over the 5-year period studied. The proportion of employees with physical disabilities was less than 2%. In terms of occupational factors, the average working hours was 40 hours or more per week in both the insured and uninsured working populations over the 5-year period. There was a decrease in working hours in 2021 and 2022. A similar pattern was observed in terms of average monthly wage, with the lowest wage recorded in 2021. There was a decreasing trend in labour union membership among the workforce, from 15.42% in 2018 to 12.02% in 2022. The number of workers with written contracts was generally less than those without contracts, and we found a declining trend from 45.70% in 2018 to 42.03% in 2022. In contrast, the number of workers employed in high risk occupations increased throughout the years (Table 1).

**Table 1.** Characteristics of insured vs uninsured working population

	2018		2019		2020		2021		2022	
Variable	Insured (*n* = 12 752)	Uninsured (*n* = 22 540)	Insured (*n* = 20 012)	Uninsured (*n* = 34 140)	Insured (*n* = 21 264)	Uninsured (*n* = 36 216)	Insured (*n* =26 684)	Uninsured (*n* = 42 820)	Insured (*n* = 19 119)	Uninsured (*n* = 32 156)
Insurance coverage rate (%)	36.13		36.95		36.99		38.39		37.29	
Individual factors										
Age (years)	38.60 ± 10.87	37.11 ± 12.80	38.72 ± 10.97	37.48 ± 12.97	38.37 ± 10.89	37.53 ± 12.88	39.03 ± 11.00	38.57 ± 13.16	38.33 ± 10.66	38.11 ± 13.03
Female gender	4587 (12.65)	7945 (21.58)	7080 (12.92)	12 036 (21.48)	7740 (13.34)	12 592 (21.08)	9987 (14.27)	13 728 (18.82)	6953 (12.62)	10 686 (20.30)
Married	9890 (26.51)	15 035 (41.39)	15 475 (27.14)	22 696 (40.55)	16 356 (27.13)	24 308 (41.11)	20 362 (28.25)	28 546 (39.87)	14 867 (27.56)	21 740 (41.90)
Education High school and above	10 704 (30.67)	8927 (24.61)	16 710 (31.38)^#^	13 967 (25.03)	18 174 (32.30)	15 150 (25.29)	22 712 (33.42)	17 761 (25.55)	16 506 (32.00)	13 771 (25.17)
Having physical disabilities	188 (0.36)	682 (1.64)	280 (0.37)	1003 (1.60)	180 (0.23)	803 (1.23)	271(0.28)	954(1.37)	190 (0.34)	754 (1.46)
Occupational factors										
Working hours (per week)	45.54 ± 11.81	44.65 ± 15.85	45.58 ± 11.57	44.48 ± 15.88	45.17 ± 11.05	44.22 ± 15.13	40.19 ± 13.49	39.53 ± 16.22	41.80 ± 12.03	38.86 ± 15.92
Duration of employment (years)	11.73 ± 9.44	8.20 ± 8.36	11.46 ± 9.35	8.37 ± 8.52	11.20 ± 9.13	8.25 ± 8.34	11.01 ± 9.53	7.88 ± 9.54	11.42 ± 9.32	9.01 ± 10.31
Income (in USD)	274.77 ± 201.24	103.18 ± 83.19	296.24 ± 238.71	118.29 ± 87.03	299.73 ± 272.07	122.69 ± 88.77	259.75 ± 199.15	105.61 ± 77.08	269.71 ± 219.70	112.19 ± 81.12
Type of employment: Employee	12 655 (36.57)	16 109 (44.91)	19 932 (37.55)	24 746 (45.07)	21 178 (37.69)	26 367 (45.23)	26 389 (39.26)	28 042 (39.06)	18 978 (37.06)	22 551 (42.97)
Field of business: Agriculture and fishery	782 (1.45)	4801 (11.84)	1794 (1.98)	7448 (11.56)	1477 (1.56)	7678 (11.62)	1964 (1.86)	10 312 (13.15)	1029 (1.32)	7714 (14.33)
Trading, reparation	939 (3.41)	2525 (8.06)	1426 (3.56)	3899 (8.24)	1630 (3.73)	4081 (7.90)	1878 (3.90)	4645 (7.49)	1415 (3.40)	3523 (7.70)
Member of labour union	5634 (13.72)	636 (1.70)	7744 (12.04)	835 (1.52)	7528 (10.86)	711 (1.04)	9237 (11.11)	829 (1.11)	6620 (10.99)	606 (1.03)
High occupational risk	1293 (3.63)	4931 (13.25)	2039 (3.76)	7584 (14.00)	2212 (3.95)	8240 (13.67)	2975 (4.63)	10 494 (14.23)	2067 (4.46)	7640 (14.78)
Having written contract	11 252 (32.27)	4947 (13.43)	17 633 (32.87)	7642 (13.59)	19 107 (33.82)	8056 (13.20)	22 803 (33.54)	7747 (9.99)	16 582 (31.95)	5829 (10.08)

*Note*: In 2018, 1 USD = 14 481 IDR. In 2019, 1 USD = 13 901 IDR. In 2020, 1 USD = 14 105 IDR. In 2021, 1 USD = 14 269 IDR. In 2022, 1 USD = 14 845 IDR.

Bivariate analysis was performed to investigate the influence of individual and occupational factors on the outcome variable of employer-sponsored insurance. Significant differences were observed between insured and uninsured workers in all individual and occupational factors. Older workers had higher coverage of employer-sponsored insurance compared with younger workers (Table 1). Similarly, employer-sponsored insurance coverage was higher among workers with high levels of education (above high school) than less educated workers. For gender, male workers were more likely to be insured than their female counterparts. Workers with physical disabilities were less likely to be insured, similar to married workers. Regarding working conditions, workers who were insured worked longer hours, were employed for longer and received higher wages than uninsured workers. Employees who were members of labour unions and had written contracts were more likely to be insured. In contrast, those in high-risk occupations were less likely to be insured, and this pattern was consistent across the 5 years examined (2018–2022).

Using logistic regression analysis (Table 2), we estimated the association between individual and occupational variables and the outcome variable—employer-sponsored health insurance (ESHI). The interaction between the variables ‘having written contract’ and ‘income at least the provincial minimum wage’ was significant (*P* < 0.05) for 2018, 2019 and 2020. However, there were only slight changes in the pseudo-R^2^ comparing the models with and without interaction, i.e. 0.001 (2018); 0.002 (2019); and 0.001 (2020), and therefore, the interaction was not included in the model above. Pseudo-R^2^ (McFadden) higher than 0.20 indicates an excellent fit. Having a written contract, earning at least the provincial minimum wage, being a member of a labour union, being employed for a duration at least 5 years and working at least 40 hours per week increased the odds of being provided with insurance by an employer. The same pattern was found from 2018 to 2022. The odds ratios for having a written contract, earning at least the provincial minimum wage and being a member of a labour union increased from 2018 to 2020 but slightly decreased in 2021 before picking up again in 2022.

**Table 2.** Factors influencing the odds of having employer-sponsored health insurance: logistic regression

	2018		2019		2020		2021		2022	
Variable	OR	95% CI	OR	95% CI	OR	95% CI	OR	95% CI	OR	95% CI
Being employed ≥5 years	1.58	1.46–1.71	1.45	1.37–1.55	1.43	1.35–1.53	1.39	1.33–1.46	1.43	1.33–1.55
Working >40 hours per week	1.35	1.24–1.47	1.38	1.29–1.48	1.32	1.23–1.41	1.35	1.29–1.42	1.29	1.20–1.39
Being member of labour union	4.61	4.06–5.24	4.52	4.04–5.06	5.56	4.94–6.27	5.23	4.80–5.70	5.05	4.33–5.89
Working in high risk jobs	0.58	0.52–0.65	0.60	0.55–0.66	0.64	0.58–0.70	0.58	0.55–0.62	0.53	0.47–0.59
Having written contract	12.81	11.69–14.03	13.39	12.46–14.38	17.09	15.91–18.35	14.22	13.56–14.91	16.99	15.72–18.37
Having physical disabilities	0.65	0.48–0.87	0.59	0.47–0.74	0.64	0.47–0.86	0.63	0.52–0.78	0.45	0.31–0.66
Earning at least the provincial minimum wage	6.58	6.04–7.16	7.029	6.574–7.515	7.14	6.68–7.63	6.13	5.83–6.45	7.43	6.84–8.07
Number of observations	35 268		54 010		57 364		69 370		50 084	
Pseudo-R^2^ (McFadden)	0.41		0.39		0.43		0.47		0.43	
Intercept	0.09		0.09		0.06		0.06		0.08	

*Notes*: A logistic regression analysis was performed after adjusting for covariates. OR, odds ratio; 95% CI, 95% confidence interval. Reference category (0): being employed <5 years, working ≤40 hours per week, not being union member, working in low/moderate risk jobs, not having written contract, not having physical disabilities, earning less than the provincial minimum wage.

To demonstrate the effect of individual and occupational factors on the provision of work accident insurance (JKK) by employers, we ran a separate logistic regression analysis (Table 3). The interaction between ‘having a written contract’ and ‘earning at least than the provincial minimum wage’ for the years 2018, 2019 and 2020 was significant (*P* < 0.05). There were no changes, however, in the pseudo-R^2^ comparing the models with and without interaction, and therefore, the interaction was not included in the model above. Pseudo-R2 (McFadden) higher than 0.20 indicates an excellent fit. We found that having a written contract, earning at least the provincial minimum wage, being a member of a labour union, working 40 hours or more per week and being employed at least 5 years increased the odds of being provided with a work accident insurance by an employer. In contrast, working in a high-risk employment and having physical disabilities decreased the odds of being provided with work accident insurance from 2018 to 2022. These results were similar to those from the analysis of the association between individual and occupational variables and the outcome variable of employer-sponsored health insurance presented in Table 1.

**Table 3.** Factors influencing the odds of having work accident insurance: logistic regression

	2018		2019		2020		2021		2022	
Variable	OR	95% CI	OR	95% CI	OR	95% CI	OR	95% CI	OR	95% CI
Being employed ≥5 years	1.53	1.41–1.66	1.53	1.44–1.64	1.43	1.35–1.52	1.17	1.10–1.25	1.32	1.23–1.42
Working >40 hours per week	1.38	1.26–1.50	1.36	1.27–1.45	1.33	1.25–1.42	1.38	1.29–1.47	1.38	1.28–1.48
Being member of labour union	4.20	3.76–4.68	4.40	4.00–4.84	4.72	4.28–5.21	2.84	2.60–3.12	3.06	2.75–3.42
Working in high risk jobs	0.84	0.74–0.94	0.83	0.76–0.91	0.88	0.80–0.96	0.86	0.79–0.94	0.77	0.70–0.86
Having written contract	11.99	10.82–13.28	11.27	10.43–12.18	14.23	13.18–15.38	12.31	11.48–13.19	12.76	11.75–13.84
Having physical disabilities	0.79	0.58–1.08	0.63	0.51–0.79	0.65	0.48–0.87	0.73	0.54–0.97	0.49	0.35–0.70
Earning at least the provincial minimum wage	5.40	4.97–5.88	6.17	5.78–6.59	5.98	5.61–6.38	4.24	3.98–4.53	5.32	4.93–5.73
Number of observations	35 268		54 010		57 364		69 402		50 084	
Pseudo-R^2^ (McFadden)	0.37		0.35		0.38		0.30		0.35	
Intercept	0.12		0.12		0.09		0.15		0.20	

*Notes*: A logistic regression analysis was performed after adjusting for covariates. OR, odds ratio; 95% CI, 95% confidence interval. Reference category (0): being employed <5 years, working ≤40 hours per week, not being union member, working in low/moderate risk jobs, not having written contract, not having physical disabilities, earning less than the provincial minimum wage.

## Discussion

Indonesia has a long way to go to achieve universal social protection for workers despite the substantial progress made over the past decades. The majority of the national labour force (over 60%), as demonstrated in our result, is not covered by either health insurance or work accident insurance. This population is vulnerable to high out-of-pocket costs in the event of work-related injury or disease. This problem is not unique to Indonesia; similar situations exist across the region and globally. In the Southeast Asian region, around 66.8% of the working population does not have access to any form of social protection (ILO, 2021a; 2021b). Similarly, nearly 70% (69.4%) of the total global workforce (about 4 billion people) is only partially covered or not covered at all by any social security benefit, including health insurance and work accident insurance (ILO, 2021c).

In Indonesia, employers are mandated to provide health insurance and work accident insurance to employees. An employer who fails to provide work accident insurance will receive a written warning, fine or forfeit certain public services, such as a business licence or building permit (GoI, 2013). Despite these sanctions, only about 16% of the total industries and enterprises in Indonesia are members of the Social Security Agency for Workers (BPJS-TK, 2021; BPS, 2022). Strengthening the enforcement of the existing laws on work accident insurance could increase compliance by companies and business entities (Ko *et al*., 2010). It must be noted that inadequate legal sanctions and law enforcement are not the only reasons for the poor compliance; several other challenges contribute to the problem. Employers might not understand the registration procedure or do not have enough resources to inform their employees about social security benefits (Boadu *et al*., 2021). Employees may not be aware of their social security rights, particularly those with limited education (Ekerdt and Hackney, 2002; Delavande and Rohwedder, 2011). In addition, workers with low education levels are more likely to engage in high risk jobs that lead to a higher risk of work-related injuries and diseases (Fujishiro *et al*., 2020). As observed in our study, more than half of the workers in high risk jobs had limited education. Building an effective labour inspection systems, educating employees about their work rights and increasing the awareness of employers on the benefits of being compliant with the labour laws are some innovative ways that should be introduced as promoted by the ILO and other countries (ILO, 2022; Syed, 2023).

This study revealed that in Indonesia, having a written contract is the most significant factor that affects the provision of ESHI to workers. An employment contract is a legally binding document between the employer and the employee that explicitly states the rights and obligations of both parties, including the right to social security (GoI, 2003a). A written contract provides a degree of certainty regarding employment status and reflects a mutually trusted relationship between the employer and employee (Guest, 2004). Providing a written employment contract is not always compulsory in every country. Across many countries in Eastern and Central Europe, it is mandatory for employers to provide a written contract to their employees (Williams and Kayaoglu, 2017). However, in countries such as Finland and Ireland in Western Europe, the provision of employment contracts is considered good practice but not legally required (Williams and Kayaoglu, 2017).

In Indonesia, employment contracts can be in written or oral format (GoI, 2021). This is applicable to all industries in the country regardless of sector or size of enterprise. However, for non-permanent workers who work for a specific time period, the employment contract must be formalized in writing (GoI, 2022b). Employers are required to provide a letter of appointment as an employee, but it is not mandatory to make a written employment contract (GoI, 2003b). This partly explains the high prevalence of workers without a written contract—averaging around 55% of the total workforce over the 5-year period examined in this study. Legalization of written employment contract is necessary to clarify the rights and obligations of workers and employers, including the social security benefits.

Our analysis also shows that workers who do not have written contracts are usually engaged in non-standard employment, defined in this study as any employment arrangements other than permanent full-time work (Polivka and Nardone, 1989). These non-standard arrangements include casual work, fixed-term contracts, part-time, on-call work, temporary agency work, labour brokering, disguised employment and self-employment (ILO). The prevalence of non-standard employment is varied across different countries and occupational categories. In Australia, the share of non-standard employment was estimated to be 55.6% in 2017 (Laß and Wooden, 2020). Meanwhile, in LMIC, the proportion could be as low as 0.8%, as shown in Russia or as high as 93.6% in Mexico among skilled agriculture workers (Apella and Zunino, 2018).

In Indonesia, about 65% of workers in the formal sector are employed under non-standard arrangements (ILO, 2016). The growing proportion of non-standard employment is concerning as it is often associated with income insecurity, lack of sufficient protection from work-related diseases and injuries and poor coverage of social security benefits (ILO, 2016). There is ample evidence linking non-standard employment with the increased risk of work-related diseases and injuries in many countries (Saloniemi and Salminen, 2010; Inoue *et al*., 2014; Maheshrengaraj and M, 2014). Studies have shown that in the absence of legal regulations, the fear of losing a job, working irregular hours and limited access to worker compensation schemes are possible reasons why workers with non-standard employment are more likely to be injured or experience work-related diseases than permanent workers (Walters and J, 2011; Probst *et al*., 2013; Foley *et al*., 2014).

During the COVID crisis, workers with non-standard employment were more prone to changes in the type of work, number of working hours and even unemployment. Over the 5-year study period, we observed an increased odds of being provided with employer-sponsored health insurance if a worker had a written contract, earned at least the provincial minimum wage and was a member of a labour union. The odds decreased in the year 2021 but bounced back in year 2022. It is likely that the dip in 2021 was influenced by the COVID-19 pandemic, given Indonesia experienced a high unemployment rate (7.1%) and poor economic growth (−2.1% of GDP) at that time (Bank, 2021; BPS, 2023). This situation was not unique to Indonesia; many countries experienced a similar slump in economic growth, with unemployment rising sharply and non-standard employment arrangements becoming more common than before (Gunn *et al*., 2022; Mitri and Sartor, 2022).

Being a member of a labour union can play a significant role in ensuring that basic work rights are protected, including being insured for occupational diseases and work accidents. Globally, 9 out of 10 union workers have better access to employer-provided insurance compared with non-union workers (Banerjee *et al*., 2021). Petach et al. also highlighted the advantage of being a union member in terms of having health insurance coverage and better access to healthcare (Petach and Wyant, 2023). Labour unions provide more power and security that allow workers to voice their rights to employers (Servais, 2022). However, in Indonesia, most labour unions represent only workers in large or medium industries and are limited in assisting workers in small to micro enterprises.

Employers in Indonesia are prohibited from paying their workers below the provincial minimum wage (GoI, 2022a). The current regulation on minimum wage applies to all employees, with the exception of those in small and micro enterprises. Employees earning less than the minimum wage could potentially be eligible for a government subsidy for national health insurance (PBI) (GoI, 2012). As seen in our study, the uninsured population from 2018 to 2022 received a monthly income ranging between IDR 1.6 million ($103) to IDR 1.9 million ($122), below the provincial minimum wage.

This study has some limitations. Firstly, the questions asked in the NLFS were not consistent across the 5-year period examined (2018–2022). For example, the surveys for 2018–2020 and 2022 had questions on employer-sponsored insurance, but the first wave survey for 2021 did not have such questions. Consequently, we used the second wave data for 2021 and the data for previous years to generate the 2021 data set (see Appendix I). Secondly, the definition of health insurance in the NLFS is not limited only to the National Health Insurance Scheme (JKN); it includes the provision of health insurance through private parties, the provision of health services at the workplace and direct reimbursement of medical costs by employers. The Central Agency on Statistics (BPS), which conducts the National Labour Survey, has provided a definition in the survey guide, but the extent to which survey respondents understood the term ‘health insurance’ remains unclear. We were unable to clarify the type of insurance respondents based on their answers as this was not recorded in the survey. Thus, in our analysis, we could not categorize the type of insurance and therefore had to analyse them as one group. Despite the above limitations, the survey comes with ready-to-use survey weights, which enables the samples to be more representative of the national workforce. Furthermore, being the first study investigating the issue of employer-sponsored insurance in Indonesia, this study could stimulate policy discussions about health insurance among workers and lead to the expansion of insurance coverage among the working population in Indonesia.

## Policy implications

This study has important implications for labour policy in Indonesia. The majority of the uninsured workforce are engaged in casual work arrangements and are less likely to be provided with health insurance or work accident insurance by their employers. Even though the Law to provide employer-sponsored health insurance already exists, there are no specific mechanisms for monitoring and promoting compliance. A guide or manual on employees’ entitlement to health insurance and work accident insurance should be developed and promoted among employers and employees to increase awareness of the employees’ right to work in a healthy and safe workplace. Furthermore, a written employment contract is essential to formalize the employees’ right to access health insurance and work accident insurance. These strategies, if implemented at the national level, could improve employers’ compliance with the regulations, which will eventually increase health insurance coverage among the Indonesian workforce.

## Conclusion

This study sought to investigate employer-sponsored health insurance and the characteristics of the uninsured working population. Overall, employer-sponsored insurance remains very low in Indonesia. Workers with written employment contracts who had worked for at least 5 years in their current employment, worked 40 hours or more a week, earned at least the provincial minimum wage and were members of a labour union were more likely to be insured. There is an urgent need to address the high rate of uninsurance among the working population in Indonesia to promote safe and healthy work conditions. Furthermore, monitoring and enforcing the Indonesian Labour Laws should be prioritized to ensure employers comply with the existing law.

## Supplementary Material

czae053_Supp

## Data Availability

The study uses data from five rounds of the Indonesian National Labour Force Survey (NLFS), 2018–2022. The NLFS is conducted every year by the Central Agency on Statistics (BPS) of Indonesia. The data are provided by the BPS and available from the Statistical Service Information System (SILASTIK) using the following link: silastik.bps.go.id. Data can be requested from BPS and interested parties are required to sign a Data Transfer Agreement.

## Reflexivity statement

The authors represent a balance of gender (two males and two females), geographical location (global north and global south) and a mix of professional expertise (occupational health experts, health economists and health system experts). The first author (LCK) is presently a doctoral student in the global north and also holds the position of academic staff in a university in the global south. The second author (MM) works exclusively in the global south in the field of occupational health. The third and fourth authors (VW and AA) are from the global north region and have vast research experience in health economics and health systems in low- and middle-income countries.
