# Supplementary material for: What explains the provision of health insurance by Indonesian employers? A trend analysis of the National Labour Force Survey 2018-2022
Source: Health Policy Plan. 2024 Jun 20;39(7):741–52. doi: 10.1093/heapol/czae053 (PMC11308606; doi:10.1093/heapol/czae053)
Supplement: czae053_Supp [file czae053_supp.zip › Revised supplementary_clean version_FINAL.docx]

**Appendix**

Calculation to estimate sample in 2021

|  | **2018** | **2019** | **2020** | **2021** |
| --- | --- | --- | --- | --- |
| **n of insured** | 12,752 | 20,012 | 21,264 | 25,027 |
| **n of uninsured** | 22,540 | 34,140 | 36,216 | 41,809 |
| **Total working population** | 35,292 | 54,152 | 57,480 | 66,837 |
| **n observation** | 129,538 | 196,385 | 203,501 | 259,695 |
| **Average increase cases of insured workers** | 0.18 |  |  |  |
| **Average increase cases of uninsured cases** | 0.15 |  |  |  |
| **Average overall increase** | 0.27 |  |  |  |

We estimated the number of insured and uninsured group in 2021 based on the average increase cases of insured and uninsured workers from year 2018 to 2020.

**Table 1. Comparison of insured versus uninsured working population across provinces in Indonesia from 2018 to 2022**

| Province | 2018*** | | 2019*** | | 2020*** | | 2021*** | | 2022*** | |
| --- | --- | --- | --- | --- | --- | --- | --- | --- | --- | --- |
|  | Insured | Uninsured | Insured | Uninsured | Insured | Uninsured | Insured | Uninsured | Insured | Uninsured |
| Aceh | 380 | 828 | 741 | 1308 | 720 | 1256 | 971 | 1871 | 556 | 1349 |
| North Sumatra | 662 | 1429 | 1212 | 2195 | 1264 | 2457 | 1504 | 2798 | 1042 | 2059 |
| West Sumatra | 404 | 751 | 585 | 1246 | 654 | 1248 | 821 | 1504 | 545 | 1245 |
| Riau | 374 | 664 | 644 | 977 | 652 | 1046 | 808 | 1246 | 565 | 975 |
| Jambi | 246 | 522 | 374 | 811 | 412 | 835 | 477 | 1060 | 366 | 752 |
| South Sumatra | 366 | 850 | 483 | 1131 | 487 | 1159 | 618 | 1567 | 536 | 1022 |
| Bengkulu | 183 | 335 | 256 | 594 | 266 | 580 | 366 | 702 | 194 | 590 |
| Lampung | 286 | 704 | 396 | 1020 | 419 | 1060 | 554 | 1447 | 414 | 1077 |
| Bangka Belitung | 148 | 346 | 236 | 484 | 280 | 497 | 306 | 578 | 256 | 467 |
| Riau Island | 265 | 196 | 399 | 387 | 523 | 429 | 562 | 493 | 439 | 339 |
| Jakarta | 484 | 416 | 769 | 513 | 796 | 568 | 771 | 608 | 714 | 357 |
| West Java | 1151 | 1819 | 1767 | 2896 | 1911 | 3081 | 2234 | 3680 | 1631 | 2865 |
| Central Java | 1144 | 2573 | 1848 | 3761 | 2001 | 4125 | 2566 | 5038 | 1758 | 3695 |
| Yogyakarta | 193 | 345 | 307 | 500 | 311 | 459 | 403 | 532 | 320 | 405 |
| East Java | 1080 | 2524 | 1596 | 4097 | 1739 | 4261 | 2336 | 4940 | 1665 | 3683 |
| Banten | 566 | 547 | 746 | 799 | 799 | 744 | 1001 | 881 | 699 | 769 |
| Bali | 372 | 614 | 561 | 874 | 601 | 987 | 626 | 954 | 526 | 780 |
| West Nusa Tenggara | 163 | 446 | 290 | 712 | 314 | 774 | 380 | 988 | 230 | 593 |
| East Nusa Tenggara | 352 | 549 | 486 | 775 | 470 | 820 | 655 | 1059 | 457 | 784 |
| West Kalimantan | 352 | 620 | 493 | 932 | 487 | 998 | 672 | 1151 | 549 | 925 |
| Central Kalimantan | 390 | 454 | 567 | 737 | 649 | 813 | 798 | 892 | 598 | 640 |
| South Kalimantan | 285 | 493 | 587 | 814 | 589 | 733 | 729 | 937 | 537 | 687 |
| East Kalimantan | 414 | 366 | 613 | 519 | 649 | 648 | 797 | 532 | 611 | 481 |
| North Kalimantan | 131 | 127 | 263 | 215 | 243 | 287 | 326 | 260 | 209 | 172 |
| North Sulawesi | 260 | 651 | 455 | 916 | 565 | 926 | 698 | 1058 | 524 | 761 |
| Central Sulawesi | 210 | 505 | 363 | 729 | 385 | 765 | 526 | 856 | 356 | 675 |
| South Sulawesi | 528 | 889 | 751 | 1310 | 782 | 1359 | 973 | 1752 | 785 | 1300 |
| Southeast Sulawesi | 226 | 423 | 333 | 594 | 373 | 636 | 484 | 851 | 385 | 579 |
| Gorontalo | 105 | 294 | 210 | 442 | 164 | 474 | 276 | 487 | 200 | 367 |
| West Sulawesi | 75 | 200 | 126 | 306 | 123 | 283 | 196 | 377 | 139 | 293 |
| Maluku | 199 | 274 | 320 | 417 | 358 | 383 | 482 | 507 | 343 | 406 |
| North Maluku | 156 | 278 | 329 | 342 | 311 | 433 | 426 | 497 | 257 | 369 |
| West Papua | 160 | 199 | 316 | 356 | 371 | 366 | 532 | 397 | 337 | 349 |
| Papua | 442 | 309 | 590 | 440 | 596 | 456 | 681 | 476 | 376 | 346 |
| TOTAL | 12,752 | 22,540 | 20,012 | 34,140 | 21,264 | 36,216 | 26,555 | 42,976 | 19,119 | 32,156 |

Note: 1 unit = 1 person.

**Table 2. Comparison of insured versus uninsured working population in Java and outside Java Island**

| Java vs Non-Java | 2018 | | 2019*** | | 2020* | | 2021*** | | 2022* | |
| --- | --- | --- | --- | --- | --- | --- | --- | --- | --- | --- |
|  | Insured | Uninsured | Insured | Uninsured | Insured | Uninsured | Insured | Uninsured | Insured | Uninsured |
| Java | 4,618 | 8,224 | 7,033 | 12,566 | 7,557 | 13,238 | 9,311 | 15,679 | 6,787 | 11,774 |
| Non-Java | 8,134 | 14,316 | 12,979 | 21,574 | 13,707 | 22,978 | 17,244 | 27,297 | 12,332 | 20,382 |

Note: *p < 0.05; ** p < 0.01; *** p < 0.001.

**Table 3. The influence of having a written contract and being union member on the likelihood of being provided insurance by employers**

|  | Insurance(1) | Insurance(2) | Insurance(3) | Insurance(4) |
| --- | --- | --- | --- | --- |
| Having a written contract | 0.533*** | 0.533*** | 0.533*** | 0.533*** |
| Being union member | 0.329*** | 0.329*** | 0.329*** | 0.329*** |
| Time fixed effects (year) | No | Yes | No | Yes |
| Regional fixed effects (province) | No | No | Yes | Yes |
| Observations | 267,929 | 267,929 | 267,929 | 267,929 |
| Adjusted R square | 0.4831 | 0.48295 | 0.46991 | 0.46863 |

Note: *p < 0.05; ** p < 0.01; *** p < 0.001. Column (1) presents results from random effect estimator. Column (2) reports fixed effects results controlling for time (year). Column (3) reports fixed effects results controlling for regional effects (province). Column (4) presents results controlling for both time and regional effects.

**Table 4. Robustness check in data set year 2018**

|  | **2018 (1)** | | **2018 (2)** | | **2018 (3)** | | **2018 (4)** | | **2018 (5)** | |
| --- | --- | --- | --- | --- | --- | --- | --- | --- | --- | --- |
| **Variable** | **OR** | **95% CI** | **OR** | **95% CI** | **OR** | **95% CI** | **OR** | **95% CI** | **OR** | **95% CI** |
| Having written contract | 12.81*** | 11.69-14.03 | 14.05*** | 12.86-15.36 | 14.12*** | 12.92-15.43 | 13.49*** | 12.38-14.69 | 13.13*** | 12.06-14.29 |
| Earning at least the provincial minimum wage | 6.58*** | 6.04-7.16 | 6.16*** | 5.68-6.69 | 6.18*** | 5.70-6.71 | 6.36*** | 5.86-6.89 | 6.72*** | 6.20-7.28 |
| Being member of labour union | 4.61*** | 4.06-5.24 | 4.68*** | 4.13-5.31 | 4.68*** | 4.13-5.31 | 4.56*** | 4.02-5.18 | 4.93*** | 4.35-5.58 |
| Being employed ≥ 5 years | 1.58*** | 1.46-1.71 | 1.56*** | 1.44-1.69 | 1.55*** | 1.43-1.68 | 1.54*** | 1.42-1.66 |  |  |
| Working > 40 hours per week | 1.35*** | 1.24-1.47 | 1.29*** | 1.19-1.41 | 1.29*** | 1.19-1.41 |  |  |  |  |
| Having physical disabilities | 0.65** | 0.48-0.87 | 0.65*** | 0.48-0.88 |  |  |  |  |  |  |
| Working in high risk jobs | 0.58*** | 0.52-0.65 |  |  |  |  |  |  |  |  |
| n of observations | 35,268 | | 35,268 | | 35,268 | | 35,268 | | 35,268 | |
| Pseudo-R^2^ (McFadden) | 0.41 | | 0.40 | | 0.40 | | 0.40 | | 0.40 | |
| Intercept | 0.09 | | 0.06 | | 0.04 | | 0.04 | | 0.05 | |

Note: *p < 0.05; ** p < 0.01; *** p < 0.001. Column (1) presents results from logistic regression analysis of all factors (i.e., having written contract, earning at least the provincial minimum wage, being member of labour union, being employed ≥ 5 years, working > 40 hours per week, having physical disabilities, working in high risk jobs). Column (2) reports results from logistic regression analysis when variable ‘working in high risk jobs’ excluded. Column (3) reports results from logistic regression analysis when variable ‘working in high risk jobs’ and ‘having physical disabilities’ excluded. Column (4) reports results from logistic regression analysis when variable ‘working in high risk jobs’, ‘having physical disabilities’, and ‘working > 40 hours per week’ excluded. Column (5) reports results from logistic regression analysis when variable ‘working in high risk jobs’, ‘having physical disabilities’, ‘working > 40 hours per week’, and ‘being employed ≥ 5 years’ excluded.

**Table 5. Robustness check in data set year 2019**

|  | **2019 (1)** | | **2019 (2)** | | **2019 (3)** | | **2019 (4)** | | **2019 (5)** | |
| --- | --- | --- | --- | --- | --- | --- | --- | --- | --- | --- |
| **Variable** | **OR** | **95% CI** | **OR** | **95% CI** | **OR** | **95% CI** | **OR** | **95% CI** | **OR** | **95% CI** |
| Having written contract | 13.39*** | 12.46-14.38 | 14.56*** | 13.58-15.62 | 14.65*** | 13.65-15.71 | 13.92*** | 13.01-14.90 | 13.60*** | 12.72-14.54 |
| Earning at least the provincial minimum wage | 7.03*** | 6.57-7.52 | 6.61*** | 6.20-7.06 | 6.63*** | 6.21-7.07 | 6.78*** | 6.35-7.23 | 7.08*** | 6.65-7.55 |
| Being member of labour union | 4.52*** | 4.04-5.06 | 4.62*** | 4.12-5.17 | 4.61*** | 4.12-5.16 | 4.50*** | 4.02-5.04 | 4.78*** | 4.28-5.34 |
| Being employed ≥ 5 years | 1.45*** | 1.37-1.55 | 1.44*** | 1.35-1.54 | 1.43*** | 1.35-1.53 | 1.41*** | 1.33-1.51 |  |  |
| Working > 40 hours per week | 1.38*** | 1.29-1.48 | 1.33*** | 1.24-1.42 | 1.33*** | 1.24-1.42 |  |  |  |  |
| Having physical disabilities | 0.59** | 0.47-0.74 | 0.59*** | 0.47-0.74 |  |  |  |  |  |  |
| Working in high risk jobs | 0.60*** | 0.55-0.66 |  |  |  |  |  |  |  |  |
| n of observations | 54,010 | | 54,010 | | 54,010 | | 54,010 | | 54,010 | |
| Pseudo-R^2^ (McFadden) | 0.39 | | 0.39 | | 0.39 | | 0.38 | | 0.38 | |
| Intercept | 0.09 | | 0.06 | | 0.04 | | 0.04 | | 0.05 | |

Note: *p < 0.05; ** p < 0.01; *** p < 0.001. Column (1) presents results from logistic regression analysis of all factors (i.e., having written contract, earning at least the provincial minimum wage, being member of labour union, being employed ≥ 5 years, working > 40 hours per week, having physical disabilities, working in high risk jobs). Column (2) reports results from logistic regression analysis when variable ‘working in high risk jobs’ excluded. Column (3) reports results from logistic regression analysis when variable ‘working in high risk jobs’ and ‘having physical disabilities’ excluded. Column (4) reports results from logistic regression analysis when variable ‘working in high risk jobs’, ‘having physical disabilities’, and ‘working > 40 hours per week’ excluded. Column (5) reports results from logistic regression analysis when variable ‘working in high risk jobs’, ‘having physical disabilities’, ‘working > 40 hours per week’, and ‘being employed ≥ 5 years’ excluded.

**Table 6. Robustness check in data set year 2020**

|  | **2020(1)** | | **2020 (2)** | | **2020(3)** | | **2020(4)** | | **2020(5)** | |
| --- | --- | --- | --- | --- | --- | --- | --- | --- | --- | --- |
| **Variable** | **OR** | **95% CI** | **OR** | **95% CI** | **OR** | **95% CI** | **OR** | **95% CI** | **OR** | **95% CI** |
| Having written contract | 17.09*** | 15.91-18.35 | 18.55*** | 17.30-19.89 | 18.64*** | 17.39-19.98 | 17.89*** | 16.72-19.13 | 17.59*** | 16.45-18.81 |
| Earning at least the provincial minimum wage | 7.14*** | 6.68-7.63 | 6.74*** | 6.32-7.19 | 6.75*** | 6.33-7.20 | 6.88*** | 6.46-7.34 | 7.16*** | 6.72-7.62 |
| Being member of labour union | 5.56*** | 4.94-6.27 | 5.67*** | 5.04-6.39 | 5.67*** | 5.03-6.38 | 5.53*** | 4.91-6.23 | 5.90*** | 5.25-6.64 |
| Being employed ≥ 5 years | 1.43*** | 1.35-1.53 | 1.43*** | 1.34-1.52 | 1.42*** | 1.34-1.51 | 1.40*** | 1.32-1.49 |  |  |
| Working > 40 hours per week | 1.32*** | 1.23-1.41 | 1.27*** | 1.19-1.36 | 1.28*** | 1.20-1.36 |  |  |  |  |
| Having physical disabilities | 0.64** | 0.47-0.86 | 0.64*** | 0.47-0.87 |  |  |  |  |  |  |
| Working in high risk jobs | 0.64*** | 0.58-0.70 |  |  |  |  |  |  |  |  |
| n of observations | 57,364 | | 57,364 | | 57,364 | | 57,364 | | 57,364 | |
| Pseudo-R^2^ (McFadden) | 0.43 | | 0.42 | | 0.42 | | 0.42 | | 0.42 | |
| Intercept | 0.06 | | 0.04 | | 0.03 | | 0.04 | | 0.03 | |

Note: *p < 0.05; ** p < 0.01; *** p < 0.001. Column (1) presents results from logistic regression analysis of all factors (i.e., having written contract, earning at least the provincial minimum wage, being member of labour union, being employed ≥ 5 years, working > 40 hours per week, having physical disabilities, working in high risk jobs). Column (2) reports results from logistic regression analysis when variable ‘working in high risk jobs’ excluded. Column (3) reports results from logistic regression analysis when variable ‘working in high risk jobs’ and ‘having physical disabilities’ excluded. Column (4) reports results from logistic regression analysis when variable ‘working in high risk jobs’, ‘having physical disabilities’, and ‘working > 40 hours per week’ excluded. Column (5) reports results from logistic regression analysis when variable ‘working in high risk jobs’, ‘having physical disabilities’, ‘working > 40 hours per week’, and ‘being employed ≥ 5 years’ excluded.

**Table 7. Robustness check in data set year 2021**

|  | **2021(1)** | | **2021(2)** | | **2021(3)** | | **2021(4)** | | **2021(5)** | |
| --- | --- | --- | --- | --- | --- | --- | --- | --- | --- | --- |
| **Variable** | **OR** | **95% CI** | **OR** | **95% CI** | **OR** | **95% CI** | **OR** | **95% CI** | **OR** | **95% CI** |
| Having written contract | 15.51*** | 14.53-16.55 | 16.58*** | 15.54-17.68 | 16.62*** | 15.59-17.73 | 16.40*** | 15.39-17.46 | 16.22*** | 15.23-17.27 |
| Earning at least the provincial minimum wage | 5.77*** | 5.37-6.20 | 5.44*** | 5.07-5.83 | 5.45*** | 5.09-5.84 | 5.50*** | 5.31-5.89 | 5.65*** | 5.28-6.05 |
| Being member of labour union | 4.72*** | 4.15-5.37 | 4.92*** | 4.33-5.59 | 4.92*** | 4.33-5.60 | 4.84*** | 4.26-5.49 | 5.04*** | 4.43-5.72 |
| Being employed ≥ 5 years | 1.28*** | 1.20-1.36 | 1.27*** | 1.19-1.35 | 1.26*** | 1.19-1.35 | 1.25*** | 1.17-1.33 |  |  |
| Working > 40 hours per week | 1.18*** | 1.11-1.26 | 1.11** | 1.04-1.18 | 1.11** | 1.04-1.18 |  |  |  |  |
| Having physical disabilities | 0.66** | 0.50-0.89 | 0.68*** | 0.50-0.91 |  |  |  |  |  |  |
| Working in high risk jobs | 0.57*** | 0.53-0.63 |  |  |  |  |  |  |  |  |
| n of observations | 69,335 | | 69,335 | | 69,335 | | 69,335 | | 69,335 | |
| Pseudo-R^2^ (McFadden) | 0.39 | | 0.38 | | 0.38 | | 0.38 | | 0.38 | |
| Intercept | 0.06 | | 0.04 | | 0.03 | | 0.03 | | 0.03 | |

Note: *p < 0.05; ** p < 0.01; *** p < 0.001. Column (1) presents results from logistic regression analysis of all factors (i.e., having written contract, earning at least the provincial minimum wage, being member of labour union, being employed ≥ 5 years, working > 40 hours per week, having physical disabilities, working in high risk jobs). Column (2) reports results from logistic regression analysis when variable ‘working in high risk jobs’ excluded. Column (3) reports results from logistic regression analysis when variable ‘working in high risk jobs’ and ‘having physical disabilities’ excluded. Column (4) reports results from logistic regression analysis when variable ‘working in high risk jobs’, ‘having physical disabilities’, and ‘working > 40 hours per week’ excluded. Column (5) reports results from logistic regression analysis when variable ‘working in high risk jobs’, ‘having physical disabilities’, ‘working > 40 hours per week’, and ‘being employed ≥ 5 years’ excluded.

**Table 8. Robustness check in data set year 2022**

|  | **2022(1)** | | **2022(2)** | | **2022(3)** | | **2022(4)** | | **2022(5)** | |
| --- | --- | --- | --- | --- | --- | --- | --- | --- | --- | --- |
| **Variable** | **OR** | **95% CI** | **OR** | **95% CI** | **OR** | **95% CI** | **OR** | **95% CI** | **OR** | **95% CI** |
| Having written contract | 16.99*** | 15.72-18.37 | 18.45*** | 17.10-19.97 | 18.58*** | 17.19-20.07 | 18.12*** | 16.80-19.54 | 17.60*** | 16.33-18.97 |
| Earning at least the provincial minimum wage | 7.43*** | 6.84-8.07 | 6.79*** | 6.28-7.35 | 6.81*** | 6.30-7.37 | 6.96*** | 6.44-7.51 | 7.26*** | 6.73-7.84 |
| Being member of labour union | 5.05*** | 4.33-5.89 | 5.29*** | 4.54-6.16 | 5.28*** | 4.53-6.16 | 5.20*** | 4.47-6.06 | 5.49*** | 4.72-6.38 |
| Being employed ≥ 5 years | 1.44*** | 1.33-1.55 | 1.43*** | 1.33-1.54 | 1.42*** | 1.32-1.53 | 1.40*** | 1.30-1.51 |  |  |
| Working > 40 hours per week | 1.29*** | 1.20-1.39 | 1.20*** | 1.12-1.30 | 1.21*** | 1.12-1.30 |  |  |  |  |
| Having physical disabilities | 0.45*** | 0.31-0.66 | 0.45*** | 0.31-0.66 |  |  |  |  |  |  |
| Working in high risk jobs | 0.53*** | 0.47-0.59 |  |  |  |  |  |  |  |  |
| n of observations | 50,084 | | 50,084 | | 50,084 | | 50,084 | | 50,084 | |
| Pseudo-R^2^ (McFadden) | 0.44 | | 0.43 | | 0.43 | | 0.43 | | 0.43 | |
| Intercept | 0.08 | | 0.05 | | 0.02 | | 0.02 | | 0.03 | |

Note: *p < 0.05; ** p < 0.01; *** p < 0.001. Column (1) presents results from logistic regression analysis of all factors (i.e., having written contract, earning at least the provincial minimum wage, being member of labour union, being employed ≥ 5 years, working > 40 hours per week, having physical disabilities, working in high risk jobs). Column (2) reports results from logistic regression analysis when variable ‘working in high risk jobs’ excluded. Column (3) reports results from logistic regression analysis when variable ‘working in high risk jobs’ and ‘having physical disabilities’ excluded. Column (4) reports results from logistic regression analysis when variable ‘working in high risk jobs’, ‘having physical disabilities’, and ‘working > 40 hours per week’ excluded. Column (5) reports results from logistic regression analysis when variable ‘working in high risk jobs’, ‘having physical disabilities’, ‘working > 40 hours per week’, and ‘being employed ≥ 5 years’ excluded.

**Table 9. Coefficient estimates of logistic regression model for employer-sponsored health insurance**

|  | **2018** | **2019** | **2020** | **2021** | **2022** |
| --- | --- | --- | --- | --- | --- |
| **Variable** | **Estimate(SE)** | **Estimate(SE)** | **Estimate(SE)** | **Estimate(SE)** | **Estimate(SE)** |
| Being employed ≥ 5 years | 0.46(0.04)*** | 0.39(0.03)*** | 0.35(0.03)*** | 0.29(0.02)*** | 0.36(0.04)*** |
| Working > 40 hours per week | 0.30(0.04)*** | 0.44(0.03)*** | 0.27(0.03)*** | 0.30(0.02)*** | 0.25(0.04)*** |
| Being member of labour union | 1.53(0.06)*** | 1.70(0.05)*** | 1.71(0.06)*** | 1.71(0.04)*** | 1.62(0.08)*** |
| Working in high risk jobs | -0.55(0.06)*** | -0.55(0.04)*** | -0.45(0.05)*** | -0.60(0.03)*** | -0.64(0.06)*** |
| Having written contract | 2.55(0.04)*** | 2.62(0.03)*** | 2.83(0.04)*** | 2.65(0.02)*** | 2.83(0.04)*** |
| Having physical disabilities | -0.43(0.15)** | -0.36(0.11)** | -0.45(0.15)** | -0.59(0.11)*** | -0.79(0.19)*** |
| Earning at least the provincial minimum wage | 1.88(0.04)*** | 2.06(0.03)*** | 1.96(0.03)*** | 1.85(0.02)*** | 2.00(0.04)*** |
| n of observations | 35,268 | 54,010 | 57,364 | 69,370 | 50,084 |
| Pseudo-R^2^ (McFadden) | 0.39 | 0.47 | 0.43 | 0.47 | 0.43 |
| Intercept | -2.41(0.17)*** | -2.73(0.12)*** | -2.87(0.17)*** | -2.74(0.12)*** | -2.56(0.21)*** |

Note: *p < 0.05; ** p < 0.01; *** p < 0.001.

**Table 10. Coefficient estimates of logistic regression model for work accident insurance**

|  | **2018** | **2019** | **2020** | **2021** | **2022** |
| --- | --- | --- | --- | --- | --- |
| **Variable** | **Estimate(SE)** | **Estimate(SE)** | **Estimate(SE)** | **Estimate(SE)** | **Estimate(SE)** |
| Being employed ≥ 5 years | 0.42(0.04)*** | 0.43(0.03)*** | 0.36(0.03)*** | 0.18(0.03)*** | 0.28(0.04)*** |
| Working > 40 hours per week | 0.32(0.04)*** | 0.30(0.03)*** | 0.29(0.03)*** | 0.32(0.03)*** | 0.32(0.04)*** |
| Being member of labour union | 1.43(0.06)*** | 1.48(0.05)*** | 1.55(0.05)*** | 1.05(0.04)*** | 1.12(0.06)*** |
| Working in high risk jobs | -0.18(0.06)** | -0.19(0.05)*** | -0.13(0.05)** | -0.21(0.04)*** | -0.26(0.05)*** |
| Having written contract | 2.48(0.05)*** | 2.42(0.04)*** | 2.66(0.04)*** | 2.47(0.03)*** | 2.55(0.04)*** |
| Having physical disabilities | -0.24(0.16) | -0.46(0.11)** | -0.43(0.15)** | -0.43(0.16)** | -0.71(0.17)*** |
| Earning at least the provincial minimum wage | 1.69(0.04)*** | 1.82(0.03)*** | 1.79(0.03)*** | 1.51(0.03)*** | 1.67 (0.04)*** |
| n of observations | 35,268 | 54,010 | 57,364 | 69,370 | 50,084 |
| Pseudo-R^2^ (McFadden) | 0.39 | 0.47 | 0.43 | 0.47 | 0.43 |
| Intercept | -2.09(0.17)*** | -2.09(0.13)*** | -2.36(0.16)*** | -1.76(0.17)*** | -1.62(0.18)*** |

Note: *p < 0.05; ** p < 0.01; *** p < 0.001.
